# Relationship between cumulative exposure to pesticides and sleep disorders among greenhouse vegetable farmers

**DOI:** 10.1186/s12889-019-6712-6

**Published:** 2019-04-03

**Authors:** Jiangping Li, Yanxing Hao, Danian Tian, Shulan He, Xian Sun, Huifang Yang

**Affiliations:** 1 Department of Epidemiology and Health Statistics, School of Public Health and Management, Ningxia Medical University, Yinchuan, 750004 China; 2 Department of Occupational and Environmental Health, School of Public Health and Management, Ningxia Medical University, 1160 Shengli Street, Xingqing District, Yinchuan, 750004 China; 3 Department of Hygienic Chemistry, School of Public Health and Management, Ningxia Medical University, Yinchuan, 750004 China

**Keywords:** Pesticides, Cumulative exposure, Sleep disorder, Farmer

## Abstract

**Background:**

In the northern region of China, many greenhouse vegetable farmers are exposed to high cumulative levels of pesticides due to long-term work in greenhouses that impacts their health. The aim of the current study was to identify the relationship between cumulative pesticide exposure and sleep disorders among farmers in Yinchuan, Northwest China.

**Methods:**

A cross-sectional study was conducted for 3 consecutive years in 2015, 2016 and 2017. Using a random sampling to select the resident teams, 1366 participants were enrolled, and information was collected via face-to-face interviews by trained investigators. Ordinal logistic, multinomial logistic and poisson logistic regression models were used to identify the associations between cumulative exposure intensity (CEI) and sleep disorders.

**Results:**

High CEI (*OR* = 1.56, 95% *CI*: 1.02–3.38) was associated with short sleep duration when compared with low CEI in the Full Model. CEI was not associated with long sleep duration. Self-rated sleep quality was associated with medium (*OR* = 1.46, 95% *CI*: 1.10–2.00) and high (*OR* = 2.50, 95% *CI*: 1.83–3.40) CEI. Similarly, having difficulty sleeping was associated with medium (*OR* = 1.52, 95% *CI*: 1.02–2.24) and high (*OR* = 1.74, 95% *CI*: 1.16–2.62) CEI. Differences in the associations by gender were also noted.

**Conclusion:**

CEI was associated with sleep disorders, and gender differences were observed. Efforts should be made by local governments to address sleep problems that result from cumulative pesticide exposure in farmers, and gender differences should be considered.

**Electronic supplementary material:**

The online version of this article (10.1186/s12889-019-6712-6) contains supplementary material, which is available to authorized users.

## Background

Sleep disturbances, a common risk factor of health conditions, affects millions of people around the world in modern society [1, 2]. Sleep disorders have also been found to be related to increased risks of all-cause morbidity and mortality [3], depression [4, 5], oral disease [6], and pain-related disease [7].

Pesticides are widely sold and used in agricultural production in China. The data according to the Food and Agriculture Organization of the United Nations (FAO) showed that the average use of pesticides per area of cropland (kg/ha) in each year in mainland China increased from 1990 to 2015. Pesticide exposure is a known risk factor of many diseases, such as cancer [8–14], asthma [15, 16], diabetes [17–19], Parkinson’s disease [20–22], leukemia [23, 24], mental diseases [25–28], and non-Hodgkin lymphoma [29] and may also be related to sleep disturbances. A case-control design study showed that increased tension, greater depression and fatigue, and more frequent symptoms of central nervous system disturbances were observed in pesticide-exposed women [30]. Although Baumert et al. revealed a positive correlation between pesticide exposure and sleep apnea among US male farmers [31], few studies have focused on the relationship between cumulative pesticide exposure (CEI) and sleep disorders.

Greenhouse planting is an important way to achieve higher crop production. More than 90% of the greenhouses in China are used for fresh vegetable or fruit production [32]; however, a previous study has reported that levels of pesticide residues in greenhouses was much higher than open-field vegetables due to the increased use of pesticides to control pests and improve crop yields [33]. Although another previous study reported that the exposure to pesticides in a greenhouse was lower than the acceptable daily intake, the threat of chronic cumulative exposure to pesticides is a health concern [34].

In the present study, 1366 independent farmers were enrolled in cross-sectional studies that were conducted in three consecutive year (2015, 2016 and 2017) to determine the prevalence of sleep disorders among greenhouse planting farmers and to evaluate the association with CEI.

## Methods

### Study setting

**Fig. 1:**
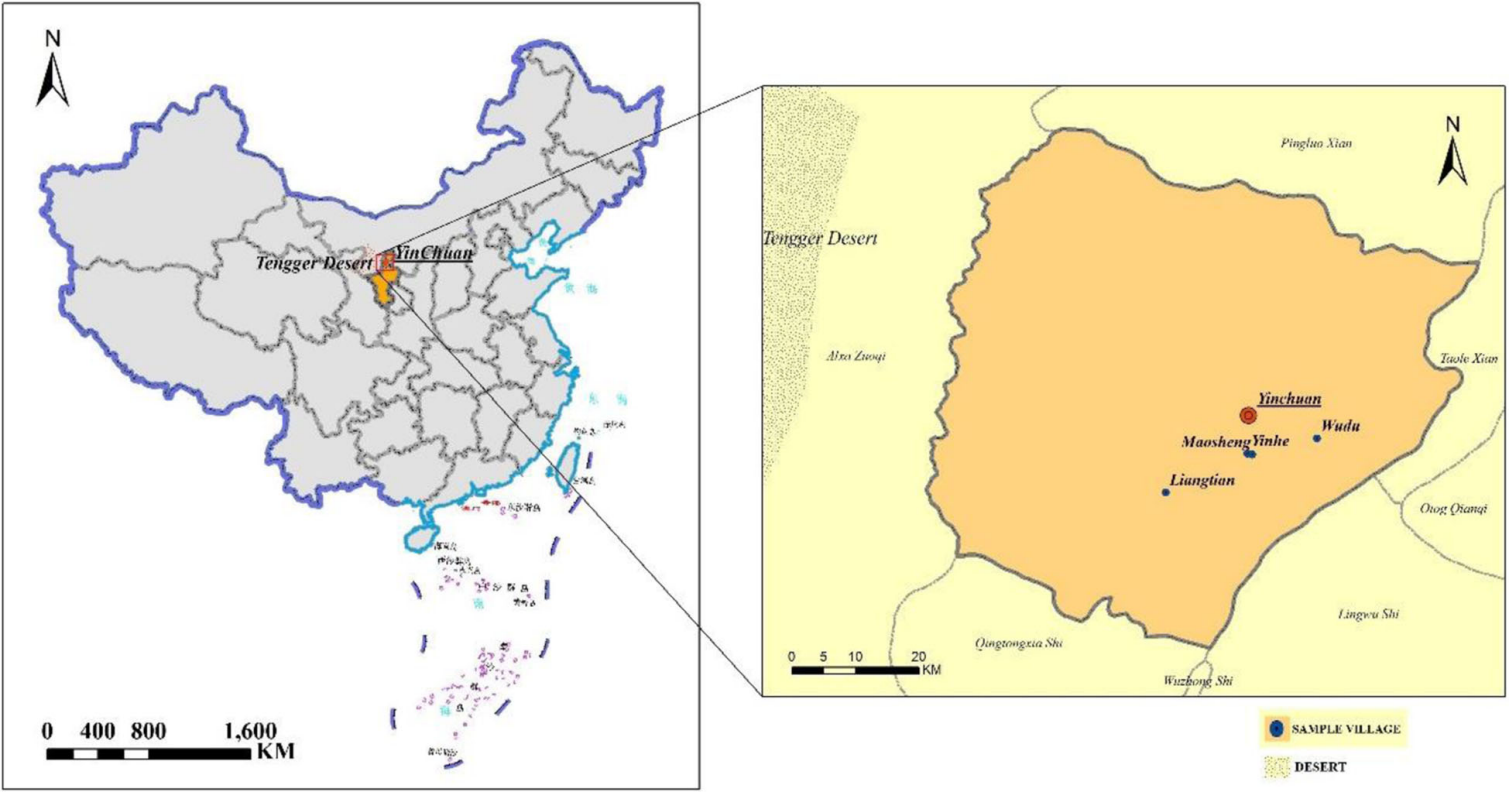
Distribution and location information of 4 villages in Yinchuan city, Ningxia, China

Yinchuan, the capital of Ningxia Hui Autonomous Region, Northwest China, is located on the border of the Tengger Desert. This region lacks water resources (a very slight declining trend in rainfall occurred from 1960 to 2006), with the per capita water resource being only one-tenth of the national average [35]. Thus, more than 60,000 ha plastic greenhouses were established to satisfy the demand of fresh vegetables for local residents in 2013, most of which were financed by the government [36]. Detailed information on the study sites are displayed in Fig. 1.

### Study design and data collection

Three cross-sectional studies were conducted from April to May in 2015, 2016, and 2017. As shown in Fig. 1, four villages (Liangtian, Maosheng, Yinhe, and Wudu) were chosen because of their long-term cooperation. In the investigation year for each village, one nonrepetitive resident team (primary resident unit in the village) was selected using a random sampling method. Six hundred questionnaires were distributed each year (150 questionnaires were sent to each resident team), and 448, 460, 458 valid, completed questionnaires were gathered in the 3 years; thus, the valid returned rate was 74.67, 76.67 and 76.33%, respectively.

The inclusion criteria for the participants were: adult greenhouse vegetable farmers who had lived at their current address at least 5 years and were engaged in vegetable planting in greenhouses more than 1 year.

Because most participants in our study were busy with farming work at the time of our investigations, verbal consent from the participating farmers was obtained prior to the interview, which helped to facilitate management and improve investigation compliance and efficiency. The design for this study was approved by the Medical Ethics Committee of Ningxia Medical University (No.2014–090). If participant disagreed to investigate, then we recorded he/she as nonresponse case on the last back of questionnaire paper. The questionnaires (Additional file 1) were completed through a face-to-face interview by a trained interviewer, and the basic personal demographic/socioeconomic information and medical history were obtained.

### Physical, psychological and social stress

The revised SRHMS (SRH measurement scale) was used to assess physical, psychological and social interaction stress for the target sample. The measurement tool was developed by Xu et al. [37, 38], and social, historical and cultural factors were considered. Forty-eight items were included in the scale, which also contained three subscales, and it has been shown to be suitable for the general population, with a high reliability, validity and sensitivity of the health status [37, 38]. The valid methods of statistical scores of each subscale were derived from previous studies [39].

### Sleep status

Sleep disturbances were identified by the following seven questions: The question, “In the last month, how long do you sleep per night average?” was used to measure sleep duration, and the response was categorized into short [40] (sleeping time less or equal to 6 h), optimal (ranging from 6 to 9 h) and long sleep duration (more than or equal to 9 h). Self-rated sleep quality was evaluated by “What do you think your sleep quality was in the last month?”, and the corresponding options were “excellent” =1, “good” =2, “worse” =3, and “much worse” =4. Hypnotic drug use, difficulty falling asleep, sleep apnea, and nightmares, suffering from disturbance of falling asleep were evaluated by “How often have you used hypnotic drugs in the last month”; “How often have you had trouble falling asleep in the last month? (failure to fall asleep within 30 min)”; “How often have you had sleep apnea in the past 30 d?”; “How often have had dreaminess or nightmares in the last 30 d?”; “How often have you suffered from trouble falling asleep in the last 30 d?”. Options for these questions were: “None” =1, “less than once a week” =2, “1 to 2 times per week” =3, “more than or equal to 3 times per week” =4.

### Cumulative pesticide exposure index

Although the information collected through the questionnaires ignored specific pesticide use and exposure levels in evaluating CEI, it would be synthesized from exposures to multiple potential pesticides.

There are several latent factors that may affect the level of exposure to pesticides, including the areas of planting, duration of work in sheds, mixing of spray pesticides, habits of spraying, equipment of protection, and habits of hygiene in personal work. CEI was evaluated using a quantitative method, with validity that had been proven by precious studies [41, 42]. CEI was calculated using an equation that, at best, provided a semi-quantitative index to represent actual pesticide exposure levels. Two algorithms were used, with several parameters in the formula, such as the habit of spraying, planting areas, and personal protective equipment (PPE), which came from the questionnaire. In the second formula, behavior in spray processes were considered as an additive predictor with mixing status and application method. This could occur because the awareness of personal protection is low among greenhouse farmers, as proven in our previous study [43], and many farmers had unhealthy habits, such as chatting, eating and drinking water during the spray process, and these habits may have increased their level of exposure to pesticides. The parameter description and assigned scores from the formula are presented in Tables 1 and 2. Finally, the CEI were placed into tertiles, lower-, medium-, and high CEI, due to the positive-skewed distribution (Additional file 2). Corresponding cut-pointing was 13.6 and 40.8.

$$ \mathrm{CEI}=\mathrm{Intensity}\times \mathrm{Hyg}\ \mathrm{Coefficient}\times \mathrm{Areas}\times \mathrm{Years} $$

$$ \mathrm{Intensity}\ \mathrm{level}=\left(\mathrm{mixing}\ \mathrm{status}+\mathrm{application}\ \mathrm{method}+\mathrm{behavior}\ \mathrm{in}\ \mathrm{spray}\right)\times \mathrm{PPE} $$

**Table 1:** Questionnaire Items, Items in the Formula and Assigned Scores

Questionnaire Item	Options and Scores	Frequency	Percentage (%)	Items in the Formula
Are you using mixed pesticides?	Never used mixing = 0	265	19.40	mixing status
	Less than 50% times = 3	396	28.99	
	More than 50% times = 9	599	43.85	
	Missing	106	7.76	
What is the way you spray pesticides?	Hand spray = 8	1112	81.41	application method
	Machine Spray = 1	113	8.27	
	Mix spray = 4	24	1.76	
	Missing	117	8.57	
In the process of spraying, do you have the following behavior?	Drink water = 3	77	5.64	behavior in spray
	Eating = 3	26	1.90	
	Smoking = 2	51	3.73	
	Chat = 1	444	32.50	
	None = 0	672	49.19	
	Missing	96	7.03	
What are the protective measures you use when using pesticides?^a^ (Multiple choice) [41]	PPE-0 = 1	482	35.29	PPE
	PPE-1 = 0.8	183	13.40	
	PPE-2 = 0.7	370	27.09	
	PPE-3 = 0.6	7	0.51	
	PPE-1 & PPE-2 = 0.5	219	16.03	
	PPE-1 & PPE-3 = 0.4	0	0.00	
	PPE-2 & PPE-3 = 0.3	6	0.44	
	PPE-1 & PPE-2 & PPE-3 = 0.1	0	0.00	
	Missing	99	7.25	
Question 1: After spraying pesticide, when do you usually clean or change into clean clothes? Question 2: What time do you have to take a shower after spraying pesticides? Question 3: What time do you wash your hands after spraying pesticide?	Question 1:			Hyg [36]
	Immediately = 1	745	54.54	
	Change the clothes that day = 2	364	26.65	
	Do not change clothes never = 3	156	11.42	
	Missing	101	7.39	
	Question 2			
	Immediately = 1	472	34.55	
	The same day = 2	584	42.75	
	Not in the same day = 3	187	13.69	
	Missing	123	9.00	
	Question 3			
	Immediately = 1	472	34.55	
	The same day = 2	584	42.75	
	Not in the same day = 3	187	13.69	
	Mssing	123	9.00	
How many years did you work in vegetable greenhouse?	1–2 years = 1	558	40.85	Years
	2–5 years = 2	154	11.27	
	5–10 years = 3	274	20.06	
	10–20 years = 4	301	22.04	
	> 20 years = 5	79	5.78	
What is the area of your greenhouse?^b^	< 1 MU^c^ = 1	23	1.68	Areas
	1–2 MU = 2	378	27.67	
	2–5 MU = 3	462	33.82	
	5–10 MU = 4	403	29.50	
	> 10 MU = 5	100	7.32	

Note: ^a^. Options were either none, masks, protective suit, protective goggles, protective gloves, or protective rubber shoes; If ‘None’ was selected, then, the assigned score was PPE-0. If one or more of these options (masks, goggles, fiber or leather gloves, old clothes) was chosen, then assign PPE-1; If one or more of these options were used along with gas masks, rubber boots, or clean protective clothing; then assign PPE-2; If rubber gloves were used, then assign PPE-3 [41]

^b^. If participants did not know the greenhouse planting area, then the following question was asked: “Do you remember the width and length of the planting area?” Then, the areas were calculated using the formula width*length

^c^. China area measurement unit, 1 MU = 666.6667 m^2^

**Table 2:** Hyg Coefficients Allocation Table [36]

Hyg Coefficient	Question 1 Score	Question 2 Score	Question 3 Score
0.2	1	1	1 or 2
0.4	1	2	1 or 2
0.6	2	1	1 or 2
	1	3	1
	1	2	3
	2	2	1 or 2
	3	1	1 or 2
0.8	2	2 or 3	2 or 3
1	3	3	3

### Statistical analysis

Questionnaire-collected data were double-entered and checked using Epidata 3.01 software and then analyzed using SPSS 24.0. Frequencies and percentages were used to report categorical variables and mean ± standard deviation (SD) for the continuous variables. The F test, Pearson, and Fisher χ2 test were conducted to estimate the differences in sociodemographic, life habit, and sleep situation variables among the different CEI groups. A poisson regression model, ordinal logistic regression model and multinomial logistic regression model (if the ordinal regression model test of parallel lines not passed then selected) were performed to assess the associations between CEI and sleep disorders; age was entered into the model as a continuous covariate.

Hypnotic drug use was recoded as a binary variable, “Yes” (constituted by < 1 times/week, 1–2 times/week, ≥3 times/week) and “No” (constituted by none) for the poisson regression model because there were only 26 participants that reported they used drugs. A multinomial logistic regression was used to identify the association between the sleep disorders and CEI, and optimal sleep duration was set as a reference. Two models were employed to test the associations between CEI and sleep disorders to identify whether the results were robust: Empty Model included CEI as an independent variable only, Full Model was adjusted by all potential confounders (include Number of family member, Gender, Ethnicity, Age, Education level, Marital status, Smoking status, Drinking status, Breakfast status, Family net income status, Number of chronic diseases, Survey year, Physical, psychological and social interaction stress). The proportional adds assumption for an ordinal logistic regression test was not passed for sleep apnea and nightmares, so the multinomial regression model was employed. *P* < 0.05 was considered statistically significant.

A stratified analysis was performed by gender due to the gender differences in physiology and psychology.

## Results

### Demographic distribution

All farmers in the present study reported that they used at least one type of pesticide to control pests and to improve the production yield. The distribution of sleep disorders was observed according to the demographic/socioeconomic characteristics and spray behavior, and the results are shown in Additional file 3. At least one sleep disorder showed a significant distribution in one of the items that was used to calculate CEI, which indicates that pesticide spray habits and behavior might be associated with sleep disorders.

The distribution of the sociodemographic, life habit, and sleep information for the CEI groups is displayed in Table 3. Overall, 1366 independent farmers were included, with 448, 460, and 458 participants surveyed in 2015, 2016, and 2017, respectively. Males accounted for 53.1% of the participants, and more than 88% were of the Han ethnic. The mean age was 46.84 ± 10.27 (range 21–79) years. Significant differences in CEI levels occurred for ethnicity, educational level, breakfast habits, family net income level, and number of chronic diseases. Hui ethnicity, high educational status, highest family net income group, two or more chronic diseases, and irregular breakfast behavior had the higher risk of high CEI (*P* < 0.05).

**Table 3:** Sociodemographic, Life habits, and Sleep Situation Characteristics by CEI Status of Plastic Greenhouse Vegetable Farmers in Yinchuan, China from 2015 to 2017

Characteristics	Sample size	CEI Level		
		Low-level (*n* = 395)	Medium-level (*n* = 424)	High-level (*n* = 411)
Number of family member (n, %)				
One person	7	1 (14.29)	3 (42.86)	3 (42.86)
Two persons	111	30 (27.03)	34 (30.63)	47 (42.34)
Three persons	209	57 (27.27)	82 (39.23)	70 (33.49)
Four and more	897	305 (34.00)	303 (33.78)	289 (32.22)
Gender (n, %)				
Male	685	212 (30.95)	239 (34.89)	234 (34.16)
Female	545	183 (33.58)	185 (33.94)	177 (32.48)
Ethnic (n, %)				
Han	1107	379 (34.24)	380 (34.33)	348 (31.44)
Hui	123	16 (13.01)	44 (35.77)	63 (51.22)
Age (Mean, SD)	1230	45.97 (10.37)	46.66 (10.18)	47.40 (9.70)
Educational level (n, %)				
No formal school education	332	12 (37.65)	127 (38.25)	80 (24.10)
Primary school	386	135 (34.97)	135 (34.97)	116 (30.05)
Junior high school	433	112 (25.87)	144 (33.26)	177 (40.88)
High school and above	79	23 (29.11)	18 (22.78)	38 (48.10)
Marital status (n, %)				
Unmarried	41	10 (24.39%)	19 (46.34%)	12 (29.27%)
Married	1166	376 (32.25%)	396 (33.96%)	394 (33.79%)
Others	23	9 (39.13%)	9 (39.13%)	5 (21.74%)
Recent smoking status (refer to past 30 days) (n, %)				
Every day	454	149 (32.82%)	162 (35.68%)	143 (31.50%)
Not every day	20	4 (20.00%)	6 (30.00%)	10 (50.00%)
Former smoker, now quit	62	20 (32.26%)	24 (38.71%)	18 (29.03%)
Never	694	222 (31.99%)	232 (33.43%)	240 (34.58%)
Drinking status (n, %)				
30 days ago,	193	58 (30.05%)	61 (31.61%)	74 (38.34%)
Within the last 30 days	275	78 (28.36%)	103 (37.45%)	94 (34.18%)
Never drinking	762	259 (33.99%)	260 (34.12%)	243 (31.89%)
Breakfast (n, %)				
Almost everyday	735	261 (35.51%)	246 (33.47%)	228 (31.02%)
Occasionally	180	38 (21.11%)	64 (35.56%)	78 (43.33%)
Few	102	27 (26.47%)	26 (25.49%)	49 (48.04%)
Never	211	67 (31.75%)	88 (41.71%)	56 (26.54%)
Family net income group (n, %)				
Quartile 1 (<¥4000)	323	117 (36.22%)	112 (34.67%)	94 (29.10%)
Quartile 2 (¥4000–¥10,000)	378	159 (42.06%)	129 (34.13%)	90 (23.81%)
Quartile 3 (¥10,000–¥20,000)	279	69 (24.73%)	108 (38.71%)	102 (36.56%)
Quartile 4 (>¥20,000)	250	50 (20.00%)	75 (30.00%)	125 (50.00%)
Number of chronic disease (n, %)				
None	1162	385 (33.13%)	398 (34.25%)	379 (32.62%)
One	49	9 (18.37%)	20 (40.82%)	20 (40.82%)
Two and more	19	1 (5.26%)	6 (31.58%)	12 (63.16%)
Survey year (n, %)				
2015	398	122 (30.65%)	140 (35.18%)	136 (34.17%)
2016	449	148 (32.96%)	131 (29.18%)	170 (37.86%)
2017	383	125 (32.64%)	153 (39.95%)	105 (27.42%)
Sleep duration (n, %)				
Short	217	54(24.88%)	73(33.64%)	90(41.47%)
Optimal	736	245(33.29%)	250(33.97%)	241(32.74%)
Long	277	96(34.66%)	101(36.46%)	80(28.88%)
Self-rated sleep quality (n, %)				
excellent	696	267 (38.36%)	254 (36.49%)	175 (25.14%)
good	342	78 (22.81%)	107 (31.29%)	157 (45.91%)
worse	166	42 (25.30%)	54 (32.53%)	70 (42.17%)
much worse	26	8 (30.77%)	9 (34.62%)	9 (34.62%)
Hypnotic drug use (n, %)^a^				
None	1204	382 (31.73%)	416 (34.55%)	406 (33.72%)
<1 times/week	11	4 (36.36%)	7 (63.64%)	0 (0.00%)
1–2 times/week	7	3 (42.86%)	1 (14.29%)	3 (42.86%)
≥ 3 times/week	8	6 (75.00%)	0 (0.00%)	2 (25.00%)
Falling asleep trouble (n, %)				
None	994	343 (34.51%)	334 (33.60%)	317 (31.89%)
<1 times/week	82	6 (7.32%)	39 (47.56%)	37 (45.12%)
1–2 times/week	64	14 (21.88%)	24 (37.50%)	26 (40.63%)
≥ 3 times/week	90	32 (35.56%)	27 (30.00%)	31 (34.44%)
Sleep apnea (n, %)				
None	1145	365 (31.88%)	395 (34.50%)	385 (33.62%)
<1 times/week	39	13 (33.33%)	14 (35.90%)	12 (30.77%)
1–2 times/week	28	11 (39.29%)	9 (32.14%)	8 (28.57%)
≥ 3 times/week	18	6 (33.33%)	6 (33.33%)	6 (33.33%)
Nightmares (n, %)				
None	868	289 (33.29%)	301 (34.68%)	278 (32.03%)
<1 times/week	120	43 (35.83%)	30 (25.00%)	47 (39.17%)
1–2 times/week	118	29 (24.58%)	45 (38.14%)	44 (37.29%)
≥ 3 times/week	124	34 (27.42%)	48 (38.71%)	42 (33.87%)
Suffer from sleep disorders (n, %)				
None	1066	346 (32.46%)	369 (34.62%)	351 (32.93%)
<1 times/week	67	19 (28.36%)	14 (20.90%)	34 (50.75%)
1–2 times/week	49	11 (22.45%)	22 (44.90%)	16 (32.65%)
≥ 3 times/week	48	19 (39.58%)	19 (39.58%)	10 (20.83%)

Note: Missing information about CEI in the calculation item resulted in a total sample size different from 1366

a: Used Fisher’s exact test

Family net income group: Calculated by family raw income minus family total expenditure in quartiles. ‘Quartile 1’ represents the lowest family finance status, while ‘Quartile 4’ is the highest family finance status

Number of chronic diseases: chronic disease information was obtained using the question “Do you have the following diseases diagnosed at the hospital?”. Options included “Hypertension”, “Coronary heart disease (CHD)”, “Hyperlipidemia”, “Stroke”, “Myocardial infarction”, “Heart failure”, “Coronary atherosclerosis”, and “Others”

¥: China Yuan (CNY)

Five sleep problems were found to be significant (*P* < 0.05) among the CEI groups from the univariate analysis (Table 3), excluding sleep apnea and nightmares. Sleep duration showed differences in distribution among the CEI groups, with 7.85, 7.74, and 7.51 average hours in the low, medium, and high CEI groups, respectively. The short, optimal and long sleep duration prevalence were 17.8, 59.4 and 22.8%, respectively. Significant differences in multi-comparisons favored high versus low CEI. Sleep duration in the high CEI group was less than in the other groups, and excellent self-rated sleep quality was observed in a smaller proportion (25.14%) of the high CEI group. Twenty-six participants reported that they had relied on medication to fall asleep in the past month; therein, 11 reported a drug use frequency of less than once a week, 7 reported 1 to 2 times per week, and 8 respondents indicated more than or equal to 3 times per week. A high frequency of medication use occurred in the high CEI group. The distribution of characteristics related to drug use are shown in Additional file 4, and three variables had significant differences. A higher proportion of having trouble falling asleep was observed in the medium and high CEI groups, and the frequency was from one to three times per week.

### Odds ratio for sleep items

Empty Model in Table 4 show that high CEI was significantly associated with short sleep duration compared with low CEI. Nonsignificant associations were observed for comparisons with the medium CEI group. There was not enough evidence to identify the relationship between CEI and long sleep duration. For self-rated sleep quality, high CEI had a tighter relationship than medium (2.46- vs. 1.37-fold likelihood, *P*-value< 0.05). A similar trend occurred in trouble falling asleep (1.82- and 1.64-fold significant likelihood, respectively). Nonsignificant differences were observed for drug use, sleep apnea, nightmares and suffered from sleep disorders in all models (data not shown for the last three items).

**Table 4:** Association between sleep issues and CEI levels from different adjusted models among plastic greenhouse vegetable farmers from Yinchuan, China, 2015–2017

Model	Sleep duration (Short vs. Optimal)^a^		Sleep duration (Long vs. Optimal) ^a^		Self-rated sleep quality^b^		Hypnotic drug use^c^		Trouble falling asleep^b^	
	OR	95% CI	OR	95% CI	OR	95% CI	IRR	95% CI	OR	95% CI
Empty Model										
Medium vs. Low	1.32	0.89–1.96	1.03	0.74–1.43	1.37	1.04–1.81	0.57	0.24–1.38	1.64	1.14–2.38
High vs. Low	1.69	1.16–2.48	0.85	0.60–1.20	2.46	1.87–3.24	0.37	0.13–1.04	1.82	1.26–2.63
Full Model										
Medium vs. Low	1.31	0.87–1.99	1.04	0.73–1.49	1.48	1.10–2.00	0.43	0.14–1.32	1.52	1.02–2.24
High vs. Low	1.56	1.02–3.38	1.11	0.76–1.64	2.5	1.83–3.40	0.49	0.19–1.24	1.74	1.16–2.62

Note: ^a^: Parameter derived from multinomial logistic regression; ^b^: Parameter derived from ordinal logistic regression; ^c^: Parameter estimated by poisson regression

*IRR*: incidence-rate ratios

The results showed robust conditions in Full Model, where the low CEI was used as the reference. Short sleep duration had a 1.56-fold significant odds relationship with high CEI. Nonsignificant associations were observed for medium CEI with short sleep duration and all CEI groups with long sleep duration. An association also found between CEI and self-rated sleep quality and having trouble falling asleep, with nearly 1.5-fold likelihood in medium CEI and 2.50- and 1.74-fold likelihood for self-rated sleep quality and trouble falling asleep in high CEI.

The sensitivity analysis was based on the sample of excluded individuals who reported using hypnotic drugs to help themselves fall asleep. The results are represented in Additional file 5, which indicates similar associations as those shown in Table 4. Stable results were carried out and further validated it.

### Stratified analysis

Stratification was according to gender, and the results are displayed in Additional file 6. For sleep duration (contained long and short sleep duration simultaneously), nonsignificant relationships with CEI were shown in the subsample of males and females. Similar results occurred for hypnotic drug use in each stratum, as there were nonsignificant associations with CEI. Both medium and high CEI were associated with self-rated sleep quality in the female, but the significant correlation was only observed with high CEI in males, for which the estimated odds ratio was close to the overall sample. In the female, having trouble falling asleep was associated with high CEI. In males, this finding only occurred in medium CEI.

## Discussion

This study revealed that adverse associations between CEI and sleep problems among greenhouse vegetable farmers. Due to the natural environmental and socioeconomical limitations in Northwest China, the local government authorities encouraged farmers to engage in jobs related to the production of greenhouse vegetables to satisfy the huge demands of the local residents for fresh vegetables. Farmers working in greenhouses would have increased CEI due to the warm, humid climate and long-term, frequent pesticide use [44], and consequently, high pesticide exposure is a risk to farmers in greenhouses that should be a public health concern.

Short sleep duration was significantly associated with high CEI, and nonsignificant results occurred for long sleep duration. One plausible reason is that most pesticides have detrimental impacts on human health, which occur via the influence of neurologic functions, such as acetylcholinesterase inhibition [45–47], and it could easily make related nerves active. Short sleep duration reflects nervous stress or disruption or highly reactive nerve cells, and exposure to pesticides might delay sleep time and reduce sleep duration. Quality of sleep [48] is an integrated index indicating the quality of sleep situations. Self-rated sleep quality was measured in the current study, and significant associations occurred with CEI at both the medium and high levels. High CEI showed a bigger likelihood to perceive worse sleep quality than medium. These findings are in line with the results of Zhao et al. [49], who reported that long-term exposure to pesticides could cause a sleep-awaking function disorder, which could lead to poor quality of sleep. Furthermore, it has also been validated by animal experiments [50] under restricted and controlled conditions.

Although exposure to carbofuran was associated with sleep apnea in 1569 U.S. male farmers in a previous report [31], a nonsignificant relationship between pesticide exposure and sleep apnea was observed in the present study. Possible reasons for this difference may be the different measurement tools used to determine sleep apnea in the two studies. The lack of a uniform concept for understanding nightmares and dreaminess might explain their nonsignificant association with CEI, as well as suffering from sleep. Furthermore, the high pain tolerance of farmers may have reduced the reflection probability of sleep disorders in local farmers. Having trouble falling asleep had significant associations with both medium and high CEI. A large portion of people that had trouble falling asleep had insomnia that due to physical pain and the use of medications. Oral exposure was a common pathway [46] in the farming spray pesticide process that tended to impact the respiratory system, meanwhile the corresponding symptoms, such as apnea and chest tightness, also occurred [51], which might influence individuals’ perception of sleep quality as well. Physical pain from respiratory diseases could also contribute to the negative relationship with sleep. Hypnotic drug use, sleep apnea and nightmare suffering from sleep disorders did not have significant relationships with CEI. The possible reason for this may be that these issues are more serious. Since the 2000s, the Chinese government has banned the sale of highly toxic pesticides and has undergone efforts to regulate the use of pesticides. That may explain the nonsignificant finding.

Differences occurred by gender for the relationship between sleep disorders and CEI. Sensitivity-perceived sleep quality was observed in the female sample and was significantly associated with both medium and high CEI level, meanwhile statistically significant differences in the male sample only occurred at the high level. This means that the quality of sleep for females was susceptible to pesticides exposure, possibly because women are more exquisite and pay attention to detail, so depending on the psychological condition, it would easily lead to a larger reduction in the quality of sleep for women than man. Interestingly, feeling trouble to sleep was associated with the medium and high levels for males and females, respectively, which was a novel finding of this research and may need further study.

Some limitations of this study need to be noted. The lack of information on specific pesticide exposures could limit the applicability of these findings. CEI may be underestimated because not all pesticide exposure came from greenhouse planting processes, as other emission sources, such as regular household exposure [52] and open environment exposure, were not considered in the current study. Memory recall bias could exist due to pesticide use and personal information recall, which might affect the precision of the results. Sleep problems were measured at one time-point; therefore, this measurement would not reflect sleep status changes and might lead to results that do not reflect the real sleep situation. Compared with a cohort study design, the fact that the current cross-sectional design could not achieve causal inference is the major limitation. Future studies should include a cohort study design and the follow-up evaluations.

## Conclusions

In conclusion, despite the above limitations, the results showed the stable effects of CEI associated with sleep problems. CEI was positively associated with short sleep duration and negatively associated with self-perceived sleep quality, and trouble falling asleep in plastic greenhouse vegetable farmers. High CEI showed a stronger correlation coefficient than medium CEI groups compared with the low exposure. Gender differences in the relationship between CEI and self-rated sleep quality and having trouble falling asleep were observed. Efforts should be made to address sleep problems due to the effects of cumulative pesticide exposure in farmers, taking into account gender differences.

## Additional files

Additional file 1: Questionnaire. (DOCX 33 kb)

Additional file 2: The distribution and describe of CEI. (DOCX 25 kb)

Additional file 3: Sleep disorders distribution in behavior variables of pesticides used characteristic and significant test (n, %). (DOCX 56 kb)

Additional file 4: Distribution of characteristic in participants between hypnotic use and nonuse. (DOCX 18 kb)

Additional file 5: Association between sleep issues and CEI levels from different adjusted models among plastic greenhouses vegetable farmers exclude sample of hypnotic drug use. (DOCX 16 kb)

Additional file 6: Subset adjusted odds ratio of sleep issues with CEI levels (reference: low CEI level) among plastic greenhouse vegetable farmers, Yinchuan, China. (DOCX 17 kb)

## Abbreviations

CEI: Cumulative Exposure Index
CI: Confidence Interval
IRR: Incidence-rate ratios
OR: Odds Ratio
