# Supplementary material for: Relationship between cumulative exposure to pesticides and sleep disorders among greenhouse vegetable farmers
Source: BMC Public Health. 2019 Apr 3;19:373. doi: 10.1186/s12889-019-6712-6 (PMC6448255; doi:10.1186/s12889-019-6712-6)
Supplement: Supplementary file 1 — Questionnaire. (DOCX 33 kb) [file 12889_2019_6712_MOESM1_ESM.docx]

Put up the questionnaire code here

**According to Article 15 of Chapter III of the "Statistics Law" of the People's Republic of China, "the investigation message belongs to individuals or families, and forbid to be leaked without the consent of the participants".**

**Health survey questionnaire for greenhouse workers in Ningxia**

| Your Name： Phone Number: | |
| --- | --- |
| Investigator Name： | Date：□□□□Year□□Month□□Day |
| Code of greenhouse：□□□□□□□□□□ | Questionnaire ID：□□□□ |

| **Part A：General Information** | | |
| --- | --- | --- |
| **A1** | How many people are there in your family? | 1.One 2. Two 3.Three 4.Four or more |
| **A2** | Your gender | 1.Male 2. Female |
| **A3** | Ethnic | 1.Han 2. Hui 3. Others |
| **A4** | Age |  |
| **A5** | Educational level | 1.No formal school education; 2. Primary school; 3. Junior high school; 4. High school and above |
| **A6** | Marital status | 1. Unmarried; 2. Married; 3. Others |
| **A7** | How long do you live in the local area? | 1.Less than 5 years; 2. 5-10years; 3.10-15 years; 4.More than 15 years |
| **A8** | What is the expenditure for your family's production last year? __________________ | |
| **A9** | What is your total income last year? ________________________ | |
| **A10** | How many diseases have you been diagnosed? ____________ | |
| **Part B: Life style and Dietary habit** | | |
| **B1** | Recent smoking status (refer to past 30 days) | 1. Every day; 2. Not every day; 3. Former smoker, now quit; 4. Never |
| **B2** | Drinking status | 1. 30 days ago; 2. Within the last 30 days; 3. Never drinking |
| **B3** | How many meals do you usually eat one day? |  |
| **B4** | Your breakfast habit？ | 1. Almost everyday; 2. Occasionally; 3. Few; 4. Never |
| **B5** | What level do you think you usually eat salt? | 1.Light; 2.Moderation; 3.Over; 99.Don’t know |
| **Part D:** **Vegetable cultivation in greenhouse** | | |
| **D1: General information** | | |
| **D1.1** | How many years did you work in vegetable greenhouse? | 1. 1-2 years; 2. 2-5 years; 3.5-10 years; 4.10-20 years; 5.>20 years |
| **D1.2** | What is the area of your greenhouse? _____MU; if no response please answer next question  Do you remember the width and length of the planting area? Length_____m, Width_____m | |
| **D1.3** | How many people in your family worked in greenhouses? | |
| **D1.6** | How many times do you work in the greenhouse every year? | 1. less than 199days 2. 200-299days 3.>300days |
| **D1.7** | What is the longest posture you keep while working in a greenhouse? | 1. Standing 2. Half squat 3. Bend the wait 4. Raise the head 5.Other ____ |
| **D1.8** | Do you eat vegetables in your own greenhouse? _____ | 1 yes 2 no |
| **D2** | **Pesticides Use** | |
| **D2.1** | Are you using mixed pesticides? | 1. Never used mixing; 2. Less than 50% times; 3. More than 50% times |
| **D2.2** | What proportion of mixed pesticides do you use? | 1. Less than half 2. More than half |
| **D2.3** | How many pesticides do you usually mix? | 1. 2 2. 3 3. 4 4. 5 5. >5 |
| **D2.4** | How long do you spray pesticides by yourself? | 1. Never 2. Less than half 3. More than half or all |
| **D 2.5** | How do you measure pesticides? | 1.Use measuring cups or other measuring instruments 2 .Use bottle cap 3. Poured with bottle directly |
| **D 2.6** | What is the way you spray pesticides? | 1. Hand spray; 2. Machine Spray; 3. Mix spray |
| **D3** | **Protection knowledge and attitude** | |
| **D3.1** | In the process of spraying, do you have the following behavior? | 1.Drink water; 2. Eating; 3. Smoking; 4. Chat; 5.None |
| **D3.2** | What are the protective measures you use when using pesticides? (Multiple choice) | 1.None; 2.Masks; 3.Protective Suit; 4.Protective Goggles; 5.Protective Gloves; 6.Protective Rubber Shoes |
| **D3.3** | Have you checked the spray device for leaks before spraying the pesticide? | 1. Yes 2 .No 3. Not paying attention |
| **D3.4** | Have you checked if the pesticide leaks when spraying pesticides? | 1. Yes 2 .No 3. Not paying attention |
| **D3.5** | After spraying pesticide, when do you usually clean or change into clean clothes? | 1. Immediately; 2. Change the clothes that day; 3. Do not change clothes never |
| **D3.6** | What time do you have to take a shower after spraying pesticides? | 1. Immediately; 2. The same day; 3. Not in the same day |
| **D3.7** | What time do you wash your hands after spraying pesticide? | 1. Immediately; 2. The same day; 3. Not in the same day |

| **Your sleep status in the last month：** | |
| --- | --- |
| In the last month, how long do you sleep per night average? | _______Hours |
| What do you think your sleep quality was in the last month? | 1=Excellent; 2=Good; 3=Worse; 4=Much worse |
| How often have you used hypnotic drugs in the last month? | 1=None; 2=Less than once a week; 3=1 to 2 times per week; 4=more than or equal to 3 times per week |
| How often have you had trouble falling asleep in the last month? (failure to fall asleep within 30 min) | 1=None; 2=Less than once a week; 3=1 to 2 times per week; 4=more than or equal to 3 times per week |
| How often have you had sleep apnea in the past 30 d? | 1=None; 2=Less than once a week; 3=1 to 2 times per week; 4=more than or equal to 3 times per week |
| How often have had dreaminess or nightmares in the last 30 d? | 1=None; 2=Less than once a week; 3=1 to 2 times per week; 4=more than or equal to 3 times per week |
| How often have you suffered from trouble falling asleep in the last 30 d? | 1=None; 2=Less than once a week; 3=1 to 2 times per week; 4=more than or equal to 3 times per week |

**Thanks For Your Cooperation**

**Have a great day!**
