# Supplementary material for: Relationship between cumulative exposure to pesticides and sleep disorders among greenhouse vegetable farmers
Source: BMC Public Health. 2019 Apr 3;19:373. doi: 10.1186/s12889-019-6712-6 (PMC6448255; doi:10.1186/s12889-019-6712-6)
Supplement: Supplementary file 2 — The distribution and describe of CEI. (DOCX 25 kb) [file 12889_2019_6712_MOESM2_ESM.docx]

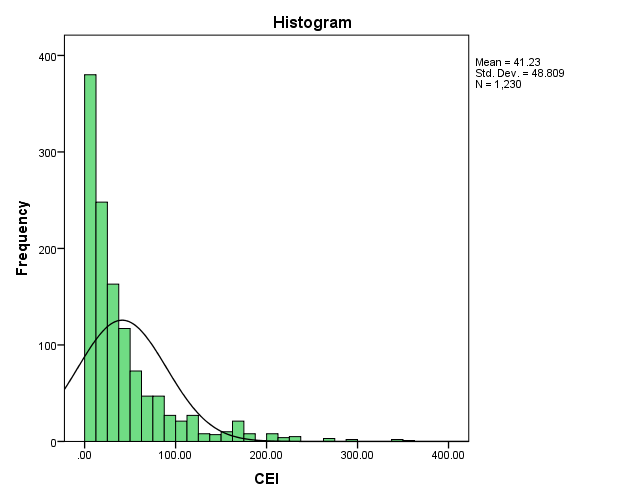


Fig. The distribution and describe of CEI

Due to missing of CEI calculate predictors, the CEI sample was 1230.
