# Supplementary material for: Relationship between cumulative exposure to pesticides and sleep disorders among greenhouse vegetable farmers
Source: BMC Public Health. 2019 Apr 3;19:373. doi: 10.1186/s12889-019-6712-6 (PMC6448255; doi:10.1186/s12889-019-6712-6)
Supplement: Supplementary file 3 — Sleep disorders distribution in behavior variables of pesticides used characteristic and significant test (n, %). (DOCX 56 kb) [file 12889_2019_6712_MOESM3_ESM.docx]

Table. Sleep disorders distribution in behavior variables of pesticides used characteristic and significant test (n, %)

| Variables | Sleep Duration | | |  | Self-rated sleep quality | | | |  | Hypnotic drug use | | | |  | Falling asleep trouble | | | |  | Sleep apnoea | | | |  | Nightmares | | | |  | Suffer from sleep disorder | | | |
| --- | --- | --- | --- | --- | --- | --- | --- | --- | --- | --- | --- | --- | --- | --- | --- | --- | --- | --- | --- | --- | --- | --- | --- | --- | --- | --- | --- | --- | --- | --- | --- | --- | --- |
|  | Short | Norm | Long |  | Excellent | Good | Worse | Much worse |  | None | <1/week | 1-2/week | >3/week |  | None | <1/week | 1-2/week | >3/week |  | None | <1/week | 1-2/week | >3/week |  | None | <1/week | 1-2/week | >3/week |  | None | <1/week | 1-2/week | >3/week |
| Mixing status^4^ |  |  |  |  |  |  |  |  |  |  |  |  |  |  |  |  |  |  |  |  |  |  |  |  |  |  |  |  |  |  |  |  |  |
| Never | 51 | 157 | 57 |  | 147 | 71 | 39 | 7 |  | 259 | 1 | 1 | 3 |  | 199 | 23 | 13 | 29 |  | 248 | 6 | 6 | 4 |  | 184 | 22 | 24 | 34 |  | 231 | 17 | 10 | 6 |
|  | 19.25% | 59.25% | 21.51% |  | 55.68% | 26.89% | 14.77% | 2.65% |  | 98.11% | 0.38% | 0.38% | 1.14% |  | 75.38% | 8.71% | 4.92% | 10.98% |  | 93.94% | 2.27% | 2.27% | 1.52% |  | 69.70% | 8.33% | 9.09% | 12.88% |  | 87.50% | 6.44% | 3.79% | 2.27% |
| Less than 50% times | 68 | 249 | 79 |  | 236 | 112 | 40 | 8 |  | 390 | 4 | 2 | 0 |  | 328 | 32 | 21 | 15 |  | 369 | 9 | 13 | 5 |  | 276 | 49 | 32 | 39 |  | 339 | 22 | 15 | 20 |
|  | 17.17% | 62.88% | 19.95% |  | 59.60% | 28.28% | 10.10% | 2.02% |  | 98.48% | 1.01% | 0.51% | 0.00% |  | 82.83% | 8.08% | 5.30% | 3.79% |  | 93.18% | 2.27% | 3.28% | 1.26% |  | 69.70% | 12.37% | 8.08% | 9.85% |  | 85.61% | 5.56% | 3.79% | 5.05% |
| More than 50% times | 103 | 348 | 148 |  | 325 | 168 | 93 | 11 |  | 582 | 6 | 4 | 5 |  | 489 | 29 | 31 | 48 |  | 554 | 25 | 9 | 9 |  | 426 | 52 | 63 | 56 |  | 520 | 29 | 25 | 23 |
|  | 17.20% | 58.10% | 24.71% |  | 54.44% | 28.14% | 15.58% | 1.84% |  | 97.49% | 1.01% | 0.67% | 0.84% |  | 81.91% | 4.86% | 5.19% | 8.04% |  | 92.80% | 4.19% | 1.51% | 1.51% |  | 71.36% | 8.71% | 10.55% | 9.38% |  | 87.10% | 4.86% | 4.19% | 3.85% |
| Application method^2,3,5,7^ |  |  |  |  |  |  |  |  |  |  |  |  |  |  |  |  |  |  |  |  |  |  |  |  |  |  |  |  |  |  |  |  |  |
| Machine | 20 | 69 | 24 |  | 60 | 33 | 18 | 2 |  | 106 | 3 | 0 | 4 |  | 92 | 8 | 5 | 8 |  | 100 | 10 | 0 | 3 |  | 89 | 8 | 9 | 7 |  | 94 | 2 | 8 | 9 |
|  | 17.70% | 61.06% | 21.24% |  | 53.10% | 29.20% | 15.93% | 1.77% |  | 93.81% | 2.65% | 0.00% | 3.54% |  | 81.42% | 7.08% | 4.42% | 7.08% |  | 88.50% | 8.85% | 0.00% | 2.65% |  | 78.76% | 7.08% | 7.96% | 6.19% |  | 83.19% | 1.77% | 7.08% | 7.96% |
| Mix | 3 | 14 | 7 |  | 12 | 3 | 5 | 3 |  | 22 | 1 | 0 | 0 |  | 17 | 2 | 0 | 4 |  | 20 | 2 | 0 | 1 |  | 12 | 2 | 5 | 4 |  | 18 | 3 | 1 | 1 |
|  | 12.50% | 58.33% | 29.17% |  | 52.17% | 13.04% | 21.74% | 13.04% |  | 95.65% | 4.35% | 0.00% | 0.00% |  | 73.91% | 8.70% | 0.00% | 17.39% |  | 86.96% | 8.70% | 0.00% | 4.35% |  | 52.17% | 8.70% | 21.74% | 17.39% |  | 78.26% | 13.04% | 4.35% | 4.35% |
| Hand | 198 | 664 | 250 |  | 631 | 312 | 147 | 22 |  | 1094 | 7 | 7 | 4 |  | 898 | 74 | 60 | 80 |  | 1041 | 28 | 29 | 14 |  | 778 | 113 | 105 | 116 |  | 969 | 63 | 41 | 39 |
|  | 17.81% | 59.71% | 22.48% |  | 56.74% | 28.06% | 13.22% | 1.98% |  | 98.38% | 0.63% | 0.63% | 0.36% |  | 80.76% | 6.65% | 5.40% | 7.19% |  | 93.62% | 2.52% | 2.61% | 1.26% |  | 69.96% | 10.16% | 9.44% | 10.43% |  | 87.14% | 5.67% | 3.69% | 3.51% |
| Behaviour in spray^3,4^ |  |  |  |  |  |  |  |  |  |  |  |  |  |  |  |  |  |  |  |  |  |  |  |  |  |  |  |  |  |  |  |  |  |
| None | 121 | 394 | 157 |  | 377 | 189 | 89 | 17 |  | 659 | 6 | 5 | 2 |  | 527 | 44 | 46 | 55 |  | 618 | 25 | 19 | 10 |  | 457 | 78 | 65 | 72 |  | 573 | 36 | 34 | 29 |
|  | 18.01% | 58.63% | 23.36% |  | 56.10% | 28.13% | 13.24% | 2.53% |  | 98.07% | 0.89% | 0.74% | 0.30% |  | 78.42% | 6.55% | 6.85% | 8.18% |  | 91.96% | 3.72% | 2.83% | 1.49% |  | 68.01% | 11.61% | 9.67% | 10.71% |  | 85.27% | 5.36% | 5.06% | 4.32% |
| Chat | 75 | 265 | 104 |  | 253 | 129 | 57 | 5 |  | 436 | 3 | 1 | 4 |  | 379 | 25 | 13 | 27 |  | 421 | 11 | 6 | 6 |  | 324 | 33 | 44 | 43 |  | 396 | 22 | 11 | 15 |
|  | 16.89% | 59.68% | 23.42% |  | 56.98% | 29.05% | 12.84% | 1.13% |  | 98.20% | 0.68% | 0.23% | 0.90% |  | 85.36% | 5.63% | 2.93% | 6.08% |  | 94.82% | 2.48% | 1.35% | 1.35% |  | 72.97% | 7.43% | 9.91% | 9.68% |  | 89.19% | 4.95% | 2.48% | 3.38% |
| Smoking | 11 | 34 | 5 |  | 31 | 7 | 10 | 2 |  | 46 | 2 | 0 | 2 |  | 36 | 7 | 2 | 5 |  | 50 | 0 | 0 | 0 |  | 38 | 6 | 3 | 3 |  | 43 | 5 | 1 | 1 |
|  | 22.00% | 68.00% | 10.00% |  | 62.00% | 14.00% | 20.00% | 4.00% |  | 92.00% | 4.00% | 0.00% | 4.00% |  | 72.00% | 14.00% | 4.00% | 10.00% |  | 100.00% | 0.00% | 0.00% | 0.00% |  | 76.00% | 12.00% | 6.00% | 6.00% |  | 86.00% | 10.00% | 2.00% | 2.00% |
| Eating/Water | 13 | 49 | 14 |  | 38 | 21 | 13 | 3 |  | 74 | 0 | 1 | 0 |  | 59 | 7 | 4 | 5 |  | 66 | 3 | 4 | 2 |  | 55 | 5 | 7 | 8 |  | 64 | 4 | 3 | 4 |
|  | 17.11% | 64.47% | 18.42% |  | 50.67% | 28.00% | 17.33% | 4.00% |  | 98.67% | 0.00% | 1.33% | 0.00% |  | 78.67% | 9.33% | 5.33% | 6.67% |  | 88.00% | 4.00% | 5.33% | 2.67% |  | 73.33% | 6.67% | 9.33% | 10.67% |  | 85.33% | 5.33% | 4.00% | 5.33% |
| PPE(Mean±sd)^1,4,5,7^ | 0.82±0.19 | .79±.19 | .78±.18 |  | .77±.19 | .81±.18 | .84±.19 | .81±.21 |  | .79±.19 | .80±.20 | .89±.20 | .85±.13 |  | .78±.19 | .81±.17 | .82±.19 | .86±.18 |  | .79±.19 | .90±.15 | .83±.16 | .84±.14 |  | .79±.19 | .78±.20 | .83±.18 | .80±.18 |  | .78±.19 | .86±.17 | .80±.18 | .87±.15 |
| Hyg1^2^ |  |  |  |  |  |  |  |  |  |  |  |  |  |  |  |  |  |  |  |  |  |  |  |  |  |  |  |  |  |  |  |  |  |
| Immediately | 127 | 450 | 168 |  | 442 | 180 | 106 | 17 |  | 729 | 7 | 5 | 4 |  | 602 | 40 | 39 | 64 |  | 688 | 29 | 19 | 9 |  | 520 | 71 | 73 | 81 |  | 648 | 35 | 31 | 31 |
|  | 17.05% | 60.40% | 22.55% |  | 59.33% | 24.16% | 14.23% | 2.28% |  | 97.85% | 0.94% | 0.67% | 0.54% |  | 80.81% | 5.37% | 5.23% | 8.59% |  | 92.35% | 3.89% | 2.55% | 1.21% |  | 69.80% | 9.53% | 9.80% | 10.87% |  | 86.98% | 4.70% | 4.16% | 4.16% |
| Change the clothes that day | 74 | 218 | 72 |  | 195 | 115 | 47 | 7 |  | 355 | 4 | 2 | 3 |  | 294 | 29 | 20 | 21 |  | 344 | 6 | 7 | 7 |  | 262 | 36 | 37 | 29 |  | 312 | 24 | 15 | 13 |
|  | 20.33% | 59.89% | 19.78% |  | 53.57% | 31.59% | 12.91% | 1.92% |  | 97.53% | 1.10% | 0.55% | 0.82% |  | 80.77% | 7.97% | 5.49% | 5.77% |  | 94.51% | 1.65% | 1.92% | 1.92% |  | 71.98% | 9.89% | 10.16% | 7.97% |  | 85.71% | 6.59% | 4.12% | 3.57% |
| Don't change clothes never | 28 | 84 | 44 |  | 68 | 55 | 23 | 6 |  | 151 | 0 | 0 | 1 |  | 117 | 15 | 6 | 14 |  | 139 | 6 | 4 | 3 |  | 103 | 15 | 13 | 21 |  | 127 | 9 | 5 | 11 |
|  | 17.95% | 53.85% | 28.21% |  | 44.74% | 36.18% | 15.13% | 3.95% |  | 99.34% | 0.00% | 0.00% | 0.66% |  | 76.97% | 9.87% | 3.95% | 9.21% |  | 91.45% | 3.95% | 2.63% | 1.97% |  | 67.76% | 9.87% | 8.55% | 13.82% |  | 83.55% | 5.92% | 3.29% | 7.24% |
| Hyg2^1,2,4,6^ |  |  |  |  |  |  |  |  |  |  |  |  |  |  |  |  |  |  |  |  |  |  |  |  |  |  |  |  |  |  |  |  |  |
| Immediately | 72 | 310 | 90 |  | 297 | 105 | 58 | 12 |  | 464 | 3 | 3 | 2 |  | 387 | 16 | 27 | 42 |  | 433 | 22 | 12 | 5 |  | 346 | 42 | 45 | 39 |  | 410 | 23 | 18 | 21 |
|  | 15.25% | 65.68% | 19.07% |  | 62.92% | 22.25% | 12.29% | 2.54% |  | 98.31% | 0.64% | 0.64% | 0.42% |  | 81.99% | 3.39% | 5.72% | 8.90% |  | 91.74% | 4.66% | 2.54% | 1.06% |  | 73.31% | 8.90% | 9.53% | 8.26% |  | 86.86% | 4.87% | 3.81% | 4.45% |
| The same day | 116 | 340 | 128 |  | 318 | 176 | 79 | 11 |  | 569 | 6 | 3 | 6 |  | 467 | 50 | 33 | 34 |  | 550 | 12 | 13 | 9 |  | 411 | 53 | 62 | 58 |  | 514 | 34 | 18 | 18 |
|  | 19.86% | 58.22% | 21.92% |  | 54.45% | 30.14% | 13.53% | 1.88% |  | 97.43% | 1.03% | 0.51% | 1.03% |  | 79.97% | 8.56% | 5.65% | 5.82% |  | 94.18% | 2.05% | 2.23% | 1.54% |  | 70.38% | 9.08% | 10.62% | 9.93% |  | 88.01% | 5.82% | 3.08% | 3.08% |
| Not in the same day | 32 | 92 | 63 |  | 86 | 65 | 32 | 4 |  | 184 | 2 | 1 | 0 |  | 149 | 17 | 5 | 16 |  | 174 | 5 | 4 | 4 |  | 119 | 27 | 12 | 29 |  | 154 | 10 | 13 | 10 |
|  | 17.11% | 49.20% | 33.69% |  | 45.99% | 34.76% | 17.11% | 2.14% |  | 98.40% | 1.07% | 0.53% | 0.00% |  | 79.68% | 9.09% | 2.67% | 8.56% |  | 93.05% | 2.67% | 2.14% | 2.14% |  | 63.64% | 14.44% | 6.42% | 15.51% |  | 82.35% | 5.35% | 6.95% | 5.35% |
| Hyg3^3^ |  |  |  |  |  |  |  |  |  |  |  |  |  |  |  |  |  |  |  |  |  |  |  |  |  |  |  |  |  |  |  |  |  |
| Immediately | 160 | 581 | 212 |  | 569 | 239 | 122 | 23 |  | 939 | 8 | 4 | 2 |  | 761 | 65 | 52 | 75 |  | 884 | 31 | 22 | 16 |  | 667 | 103 | 92 | 91 |  | 827 | 51 | 37 | 38 |
|  | 16.79% | 60.97% | 22.25% |  | 59.71% | 25.08% | 12.80% | 2.41% |  | 98.53% | 0.84% | 0.42% | 0.21% |  | 79.85% | 6.82% | 5.46% | 7.87% |  | 92.76% | 3.25% | 2.31% | 1.68% |  | 69.99% | 10.81% | 9.65% | 9.55% |  | 86.78% | 5.35% | 3.88% | 3.99% |
| The same day | 51 | 129 | 51 |  | 112 | 80 | 36 | 3 |  | 219 | 3 | 3 | 6 |  | 188 | 17 | 11 | 15 |  | 215 | 8 | 6 | 2 |  | 170 | 12 | 24 | 25 |  | 200 | 16 | 8 | 7 |
|  | 22.08% | 55.84% | 22.08% |  | 48.48% | 34.63% | 15.58% | 1.30% |  | 94.81% | 1.30% | 1.30% | 2.60% |  | 81.39% | 7.36% | 4.76% | 6.49% |  | 93.07% | 3.46% | 2.60% | 0.87% |  | 73.59% | 5.19% | 10.39% | 10.82% |  | 86.58% | 6.93% | 3.46% | 3.03% |
| Not in the same day | 8 | 31 | 18 |  | 18 | 27 | 11 | 1 |  | 57 | 0 | 0 | 0 |  | 52 | 1 | 2 | 2 |  | 56 | 0 | 1 | 0 |  | 37 | 7 | 3 | 10 |  | 49 | 0 | 4 | 4 |
|  | 14.04% | 54.39% | 31.58% |  | 31.58% | 47.37% | 19.30% | 1.75% |  | 100.00% | 0.00% | 0.00% | 0.00% |  | 91.23% | 1.75% | 3.51% | 3.51% |  | 98.25% | 0.00% | 1.75% | 0.00% |  | 64.91% | 12.28% | 5.26% | 17.54% |  | 85.96% | 0.00% | 7.02% | 7.02% |
| Planting Years^1,2,3,4,5,6^ |  |  |  |  |  |  |  |  |  |  |  |  |  |  |  |  |  |  |  |  |  |  |  |  |  |  |  |  |  |  |  |  |  |
| 1-2 years | 7 | 12 | 4 |  | 14 | 4 | 4 | 1 |  | 20 | 2 | 1 | 0 |  | 19 | 0 | 1 | 3 |  | 21 | 0 | 2 | 0 |  | 19 | 3 | 0 | 1 |  | 20 | 2 | 1 | 0 |
|  | 30.43% | 52.17% | 17.39% |  | 60.87% | 17.39% | 17.39% | 4.35% |  | 86.96% | 8.70% | 4.35% | 0.00% |  | 82.61% | 0.00% | 4.35% | 13.04% |  | 91.30% | 0.00% | 8.70% | 0.00% |  | 82.61% | 13.04% | 0.00% | 4.35% |  | 86.96% | 8.70% | 4.35% | 0.00% |
| 2-5 years | 62 | 221 | 95 |  | 256 | 70 | 44 | 8 |  | 376 | 0 | 2 | 0 |  | 330 | 9 | 15 | 24 |  | 360 | 12 | 5 | 1 |  | 291 | 26 | 25 | 36 |  | 342 | 14 | 7 | 15 |
|  | 16.40% | 58.47% | 25.13% |  | 67.72% | 18.52% | 11.64% | 2.12% |  | 99.47% | 0.00% | 0.53% | 0.00% |  | 87.30% | 2.38% | 3.97% | 6.35% |  | 95.24% | 3.17% | 1.32% | 0.26% |  | 76.98% | 6.88% | 6.61% | 9.52% |  | 90.48% | 3.70% | 1.85% | 3.97% |
| 5-10 years | 63 | 259 | 140 |  | 281 | 117 | 54 | 7 |  | 445 | 5 | 2 | 7 |  | 384 | 24 | 18 | 33 |  | 422 | 8 | 16 | 13 |  | 313 | 58 | 44 | 44 |  | 383 | 25 | 25 | 26 |
|  | 13.64% | 56.06% | 30.30% |  | 61.22% | 25.49% | 11.76% | 1.53% |  | 96.95% | 1.09% | 0.44% | 1.53% |  | 83.66% | 5.23% | 3.92% | 7.19% |  | 91.94% | 1.74% | 3.49% | 2.83% |  | 68.19% | 12.64% | 9.59% | 9.59% |  | 83.44% | 5.45% | 5.45% | 5.66% |
| 10-20 years | 95 | 252 | 56 |  | 172 | 141 | 74 | 15 |  | 396 | 3 | 2 | 1 |  | 291 | 47 | 24 | 40 |  | 373 | 17 | 8 | 4 |  | 259 | 37 | 50 | 56 |  | 353 | 21 | 13 | 15 |
|  | 23.57% | 62.53% | 13.90% |  | 42.79% | 35.07% | 18.41% | 3.73% |  | 98.51% | 0.75% | 0.50% | 0.25% |  | 72.39% | 11.69% | 5.97% | 9.95% |  | 92.79% | 4.23% | 1.99% | 1.00% |  | 64.43% | 9.20% | 12.44% | 13.93% |  | 87.81% | 5.22% | 3.23% | 3.73% |
| >20 years | 16 | 68 | 16 |  | 45 | 42 | 12 | 1 |  | 99 | 1 | 0 | 0 |  | 73 | 9 | 12 | 6 |  | 92 | 5 | 1 | 2 |  | 72 | 13 | 9 | 6 |  | 83 | 9 | 6 | 2 |
|  | 16.00% | 68.00% | 16.00% |  | 45.00% | 42.00% | 12.00% | 1.00% |  | 99.00% | 1.00% | 0.00% | 0.00% |  | 73.00% | 9.00% | 12.00% | 6.00% |  | 92.00% | 5.00% | 1.00% | 2.00% |  | 72.00% | 13.00% | 9.00% | 6.00% |  | 83.00% | 9.00% | 6.00% | 2.00% |
| Planting area^1,2,4^ |  |  |  |  |  |  |  |  |  |  |  |  |  |  |  |  |  |  |  |  |  |  |  |  |  |  |  |  |  |  |  |  |  |
| <1 MU | 86 | 309 | 163 |  | 356 | 124 | 64 | 13 |  | 539 | 8 | 4 | 6 |  | 470 | 20 | 22 | 45 |  | 513 | 18 | 13 | 13 |  | 405 | 45 | 51 | 56 |  | 476 | 26 | 21 | 34 |
|  | 15.41% | 55.38% | 29.21% |  | 63.91% | 22.26% | 11.49% | 2.33% |  | 96.77% | 1.44% | 0.72% | 1.08% |  | 84.38% | 3.59% | 3.95% | 8.08% |  | 92.10% | 3.23% | 2.33% | 2.33% |  | 72.71% | 8.08% | 9.16% | 10.05% |  | 85.46% | 4.67% | 3.77% | 6.10% |
| 1-2 MU | 23 | 104 | 27 |  | 84 | 45 | 21 | 4 |  | 153 | 0 | 1 | 0 |  | 122 | 11 | 7 | 14 |  | 144 | 4 | 6 | 0 |  | 101 | 18 | 18 | 17 |  | 130 | 8 | 10 | 6 |
|  | 14.94% | 67.53% | 17.53% |  | 54.55% | 29.22% | 13.64% | 2.60% |  | 99.35% | 0.00% | 0.65% | 0.00% |  | 79.22% | 7.14% | 4.55% | 9.09% |  | 93.51% | 2.60% | 3.90% | 0.00% |  | 65.58% | 11.69% | 11.69% | 11.04% |  | 84.42% | 5.19% | 6.49% | 3.90% |
| 2-5 MU | 55 | 170 | 49 |  | 131 | 90 | 45 | 6 |  | 269 | 0 | 1 | 2 |  | 211 | 27 | 20 | 14 |  | 252 | 8 | 8 | 4 |  | 181 | 38 | 27 | 26 |  | 235 | 17 | 9 | 11 |
|  | 20.07% | 62.04% | 17.88% |  | 48.16% | 33.09% | 16.54% | 2.21% |  | 98.90% | 0.00% | 0.37% | 0.74% |  | 77.57% | 9.93% | 7.35% | 5.15% |  | 92.65% | 2.94% | 2.94% | 1.47% |  | 66.54% | 13.97% | 9.93% | 9.56% |  | 86.40% | 6.25% | 3.31% | 4.04% |
| 5-10 MU | 59 | 190 | 52 |  | 159 | 89 | 44 | 8 |  | 296 | 3 | 1 | 0 |  | 227 | 29 | 17 | 27 |  | 283 | 11 | 5 | 1 |  | 209 | 28 | 26 | 37 |  | 268 | 16 | 11 | 5 |
|  | 19.60% | 63.12% | 17.28% |  | 53.00% | 29.67% | 14.67% | 2.67% |  | 98.67% | 1.00% | 0.33% | 0.00% |  | 75.67% | 9.67% | 5.67% | 9.00% |  | 94.33% | 3.67% | 1.67% | 0.33% |  | 69.67% | 9.33% | 8.67% | 12.33% |  | 89.33% | 5.33% | 3.67% | 1.67% |
| >10 MU=5 | 20 | 39 | 20 |  | 38 | 26 | 14 | 1 |  | 79 | 0 | 0 | 0 |  | 67 | 2 | 4 | 6 |  | 76 | 1 | 0 | 2 |  | 58 | 8 | 6 | 7 |  | 72 | 4 | 1 | 2 |
|  | 25.32% | 49.37% | 25.32% |  | 48.10% | 32.91% | 17.72% | 1.27% |  | 100.00% | 0.00% | 0.00% | 0.00% |  | 84.81% | 2.53% | 5.06% | 7.59% |  | 96.20% | 1.27% | 0.00% | 2.53% |  | 73.42% | 10.13% | 7.59% | 8.86% |  | 91.14% | 5.06% | 1.27% | 2.53% |

Note: Superscript symbol number: 1 to 7 represent sleep duration, Self-rated sleep quality, Hypnotic drug use, Falling asleep trouble, Sleep apnoea, Nightmares, Suffer from sleep disorder in the variable exist statistic significant distribution, *P*<0.05; Hyg1 to Hyg3 represent Question 1: After spraying pesticide, when do you usually clean or change into clean clothes? Question 2: What time do you have to take a shower after spraying pesticides? Question 3: What time do you wash your hands after spraying pesticide?, respectively.
