# Supplementary material for: Relationship between cumulative exposure to pesticides and sleep disorders among greenhouse vegetable farmers
Source: BMC Public Health. 2019 Apr 3;19:373. doi: 10.1186/s12889-019-6712-6 (PMC6448255; doi:10.1186/s12889-019-6712-6)
Supplement: Supplementary file 4 — Distribution of characteristic in participants between hypnotic use and nonuse. (DOCX 18 kb) [file 12889_2019_6712_MOESM4_ESM.docx]

Table. Distribution of characteristic in participants between hypnotic use and nonuse

| Variables | Hypnotic drug didn’t used | Hypnotic drug use | *P* |
| --- | --- | --- | --- |
| Number of family member (n, %) |  |  | 0.003 |
| One | 7(100.0%) | 0(0.0%) |  |
| Two | 122(94.6%) | 7(5.4%) |  |
| Three | 224(96.6%) | 8(3.4%) |  |
| Four or more | 975(98.9%) | 11(1.1%) |  |
| Gender (n, %) |  |  | 0.322 |
| Male | 712(98.5%) | 11(1.5%) |  |
| Female | 624(97.7%) | 15(2.3%) |  |
| Ethnic (n, %) |  |  | 0.760 |
| Han | 1182(98.0%) | 24(2.0%) |  |
| Hui | 154(98.7%) | 2(1.3%) |  |
| Educational level (n, %) |  |  | 0.599 |
| No formal school education | 366(98.1%) | 7(1.9%) |  |
| Primary school | 425(97.9%) | 9(2.1%) |  |
| Junior high school | 455(97.8%) | 10(2.2%) |  |
| High school and above | 90(100.0%) | 0(0.0%) |  |
| Marital status (n, %) |  |  | 0.013 |
| Unmarried | 38(90.5%) | 4(9.5%) |  |
| Married | 1270(98.3%) | 22(1.7%) |  |
| Others | 28(100.0%) | 0(0.0%) |  |
| Smoking |  |  | 0.075 |
| Recent smoking status (n, %) | 482(98.8%) | 6(1.2%) |  |
| Every day | 20(90.9%) | 2(9.1%) |  |
| Not every day | 61(98.4%) | 1(1.6%) |  |
| Former smoker, now quit | 773(97.8%) | 17(2.2%) |  |
| Drinking status (n, %) |  |  | 0.374 |
| 30 days ago, | 207(99.0%) | 2(1.0%) |  |
| Within the last 30 days | 286(97.3%) | 8(2.7%) |  |
| Never drinking | 842(98.1%) | 16(1.9%) |  |
| Breakfast (n, %) |  |  | 0.134 |
| Almost everyday | 795(98.0%) | 16(2.0%) |  |
| Occasionally | 197(99.0%) | 2(1.0%) |  |
| Few | 115(100.0%) | 0(0.0%) |  |
| Never | 227(96.6%) | 8(3.4%) |  |
| Number of chronic disease (n, %) |  |  | 0.022 |
| None | 1259(98.4%) | 21(1.6%) |  |
| One | 56(93.3%) | 4(6.7%) |  |
| Two and more | 21(95.5%) | 1(4.5%) |  |
| AGE | 46.88±10.22 | 45.27±12.99 | 0.344* |

Note: *, *P*-value calculated from Mann-Whitney Test. The rest of *P*-value derived from Chi-square or Fisher exact test.
