# Supplementary material for: Relationship between cumulative exposure to pesticides and sleep disorders among greenhouse vegetable farmers
Source: BMC Public Health. 2019 Apr 3;19:373. doi: 10.1186/s12889-019-6712-6 (PMC6448255; doi:10.1186/s12889-019-6712-6)
Supplement: Supplementary file 5 — Association between sleep issues and CEI levels from different adjusted models among plastic greenhouses vegetable farmers exclude sample of hypnotic drug use. (DOCX 16 kb) [file 12889_2019_6712_MOESM5_ESM.docx]

Table. Association between sleep issues and CEI levels from different adjusted models among plastic greenhouses vegetable farmers exclude sample of hypnotic drug use

| Model | Sleep duration (Short vs. Optimal)† | |  | Sleep duration (Long vs. Optimal) † | |  | Self-rated sleep quality⸸ | |  | Falling asleep trouble⸸ | |
| --- | --- | --- | --- | --- | --- | --- | --- | --- | --- | --- | --- |
|  | OR | 95% CI |  | OR | 95%CI |  | OR | 95% CI |  | OR | 95% CI |
| **Empty Model** |  |  |  |  |  |  |  |  |  |  |  |
| Medium vs. Low | 1.48 | 0.98-2.23 |  | 1.06 | 0.76-1.48 |  | 1.43 | 1.08-1.91 |  | 1.97 | 1.33-2.92 |
| High vs. Low | 1.95 | 1.31-2.91 |  | 0.88 | 0.62-1.24 |  | 2.65 | 2.00-3.50 |  | 2.24 | 1.52-3.31 |
| **Full Model** |  |  |  |  |  |  |  |  |  |  |  |
| Medium vs. Low | 1.48 | 0.95-2.27 |  | 1.07 | 0.75-1.53 |  | 1.57 | 1.16-2.14 |  | 1.84 | 1.38-3.10 |
| High vs. Low | 1.83 | 1.18-2.84 |  | 1.14 | 0.77-1.69 |  | 2.74 | 2.00-3.75 |  | 2.13 | 1.21-2.8 |

Note:

†: Parameter derived from multinomial logistic regression; ⸸: Parameter derived from ordinal logistic regression;

IRR: incidence-rate ratios
