# Supplementary material for: Relationship between cumulative exposure to pesticides and sleep disorders among greenhouse vegetable farmers
Source: BMC Public Health. 2019 Apr 3;19:373. doi: 10.1186/s12889-019-6712-6 (PMC6448255; doi:10.1186/s12889-019-6712-6)
Supplement: Supplementary file 6 — Subset adjusted odds ratio of sleep issues with CEI levels (reference: low CEI level) among plastic greenhouse vegetable farmers, Yinchuan, China. (DOCX 17 kb) [file 12889_2019_6712_MOESM6_ESM.docx]

Table. Subset adjusted odds ratio of sleep issues with CEI levels (reference: low CEI level) among plastic greenhouse vegetable farmers, Yinchuan, China

| Gender | Sleep duration (Short vs. Optimal)† | |  | Sleep duration (Long vs. Optimal)† | |  | Self-rated sleep quality⸸ | |  | Hypnotic drug use‡ | |  | Falling asleep trouble⸸ | |
| --- | --- | --- | --- | --- | --- | --- | --- | --- | --- | --- | --- | --- | --- | --- |
|  | OR | 95%CI |  | OR | 95%CI |  | OR | 95%CI |  | IRR | 95%CI |  | OR | 95%CI |
| ***Male*** |  |  |  |  |  |  |  |  |  |  |  |  |  |  |
| Medium vs. Low | 1.35 | 0.77-2.37 |  | 1.29 | 0.79-2.11 |  | 1.2 | 0.79-1.81 |  | 0.83 | 0.15-4.67 |  | 1.98 | 1.08-3.62 |
| High vs. Low | 1.53 | 0.86-2.74 |  | 1.27 | 0.75-2.15 |  | 1.68 | 1.10-2.58 |  | 0.34 | 0.05-2.23 |  | 1.81 | 0.96-3.44 |
| ***Female*** |  |  |  |  |  |  |  |  |  |  |  |  |  |  |
| Medium vs. Low | 1.32 | 0.69-2.51 |  | 0.84 | 0.48-1.46 |  | 2 | 1.27-3.14 |  | 0.18 | 0.02-1.86 |  | 1.28 | 0.74-2.21 |
| High vs. Low | 1.42 | 0.74-2.75 |  | 0.87 | 0.47-1.63 |  | 3.92 | 2.44-6.31 |  | 1 | 0.29-3.44 |  | 1.91 | 1.08-3.38 |

†: Parameter derived from multinomial logistic regression; ⸸: Parameter derived from ordinal logistic regression; ‡: Parameter estimated by Poisson regression.

IRR: incidence-rate ratios
